# A bibliometric analysis of research in the field of forensic psychiatry

**DOI:** 10.1192/j.eurpsy.2024.345

**Published:** 2024-08-27

**Authors:** M. M. M. Kaggwa

**Affiliations:** Department of Psychiatry and Behavioral Neurosciences, mcmaster University, Hamilton, Canada

## Abstract

**Introduction:**

Forensic psychiatry is a subspeciality that encompasses applying scientific and clinical expertise in legal contexts. As a field of psychiatry, forensic psychiatry has continued to evolve in various jurisdictions. Several journal publications continue to highlight the contributions and works of various psychiatry researchers in this area on scientific development and trends in practice. However, a quantitative assessment of these publications using a bibliometric analysis has yet to be done. Thus, the present study.

**Objectives:**

Provide a qualitative assessment of the bibliometrics of peer-reviewed research in forensic psychiatry.

**Methods:**

In this bibliometric analysis, we used Web of Science (the most frequently used database) to identify research articles in forensic psychiatry from inception to December 2023. Analysis was done using citespace and VOSviewer software.

**Results:**

Five thousand six hundred ninety articles were identified with 115 countries, 4144 institutions and universities, and 1660 authors. The articles were published in 1022 journals (most are specific to the field), and 4707 unique keywords were used to identify relevant articles. Risk assessments, violence, recidivism, psychopathy, and schizophrenia are the main areas researched. Sixteen funding agencies have funded ten or more articles in the field. The studies were mainly from high-income countries and a relatively scant number from low-income countries, especially African countries. Publications with themes on risk assessment tools – such as the HCR-20- appeared predominant across the analyzed publications.

**Conclusions:**

Research in forensic psychiatry has continued to grow over time. While many jurisdictions across the globe have embraced the field, more effort is needed to promote forensic psychiatry and research in low- and middle-income countries (LMICs). The themes or keywords that emerged from the publications included in this analysis suggest that forensic psychiatry mainly deals with offenders with schizophrenia or psychopathy.

**Disclosure of Interest:**

None Declared

